# Comparison of cellular, mechanical, and optical properties of different polymers for corneal tissue engineering

**DOI:** 10.22038/ijbms.2025.85468.18477

**Published:** 2025

**Authors:** Sepehr Zamani, Javad Sadeghi, Mohammad Kamalabadi-Farahani, Seyede Nazanin Aghayan, Zohreh Arabpour, Ali R Djalilian, Majid Salehi

**Affiliations:** 1 Student Research Committee, School of Medicine, Shahroud University of Medical Sciences, Shahroud, Iran; 2 Eye Research Center, Mashhad University of Medical Sciences, Mashhad, Iran; 3 Department of Tissue Engineering, School of Medicine, Shahroud University of Medical Sciences, Shahroud, Iran; 4 Department of Ophthalmology and Visual Sciences, University of Illinois at Chicago, IL, USA; 5 Regenerative Medicine Research Center, Shahroud University of Medical Sciences, Shahroud, Iran

**Keywords:** Biocompatible polymers, Cornea, Mesenchymal stem cells, Solvent casting, Transparent scaffolds

## Abstract

**Objective(s):**

The invention of corneal tissue engineering is essential for vision due to the lack of effective treatments and donated corneas. Finding the right polymer is crucial for reducing inflammation, ensuring biocompatibility, and mimicking natural cornea properties.

**Materials and Methods:**

In this study, solvent casting and physical crosslinking (freeze-thaw cycles) were used to fabricate polymeric scaffolds of Polyvinyl alcohol, alginate, gelatin, carboxymethyl chitosan, carboxymethyl cellulose, polyacrylic acid, polyvinyl pyrrolidone, and their combinations. The mechanical evaluation of scaffolds for tension and suture ability was conducted. Biodegradability, swelling, water vapor, bacterial permeability, anti-inflammatory properties, blood compatibility, Blood Clotting Index (BCI), pH alterations, and cell compatibility with human Mesenchymal Stem cells (MSCs) were investigated with MTT. The hydrophilicity of the samples and the ability to adhere to surfaces were also compared with the contact angle and adhesive test, respectively. Finally, quantitative and qualitative analysis was used to check the transparency of the samples.

**Results:**

The mechanical strength of polyvinyl alcohol and polyvinyl pyrrolidone samples was highest, showing good suture ability. All samples had blood compatibility below 5% and cell compatibility above 75%. Polyvinyl alcohol was the most transparent at around 93%. Carboxymethyl chitosan effectively inhibited bacterial permeability, while its anti-inflammatory potential showed no significant difference.

**Conclusion:**

This study aims to choose the best polymer composition for corneal tissue engineering. The selection depends on the study’s goals, like mechanical strength or transparency. Comparing polymers across different dimensions provides better insight for polymer selection.

## Introduction

The eye is a highly complex and relatively isolated organ within the human body, comprising two primary segments: the anterior and posterior regions. These segments are equipped with multiple protective barriers and systems designed to prevent foreign substances from penetrating the eyeball. Key protective structures include the cornea, eyelids, nasolacrimal drainage pathway, blood-retinal barrier, and blood-aqueous barrier. The cornea, with an average thickness of approximately 500 µm, exhibits unique properties such as clarity, elasticity, and transparency, which are critical for maintaining vision and protecting the internal structures of the eye (1). Due to the eye’s intricate anatomy and physiological barriers, drug delivery to both the anterior and posterior segments poses significant challenges. Globally, a substantial number of patients have legal blindness caused by corneal diseases or injuries. Corneal blindness can result from various factors, including microbial infections, dystrophies, traumatic injuries, inflammatory conditions, and degenerative diseases. In many cases, partial or full-thickness corneal transplantation (keratoplasty) remains the only viable treatment option (2). Among the most common corneal disorders are dry eye syndrome, stromal dystrophy, corneal ulcers, keratoconus, endothelial dysfunction, severe thermal or chemical burns, cicatricial pemphigoid, Stevens-Johnson syndrome, fibrosis, and wound contracture. Genetic disorders, aging, and environmental damage are significant contributing factors to the development of corneal pathologies. These conditions underscore the importance of advancing research and therapeutic strategies to address the complexities of corneal health and disease (3). 

Conventional treatments for corneal conditions involve contact lenses, artificial tears, and transplantation of donor tissue (4). Keratoplasty, commonly termed corneal transplantation, involves any surgical technique in which a human donor’s whole or partial cornea is transplanted into the recipient’s eye. It serves as the main, initial treatment for mending a harmed cornea. However, even though corneal transplants are now a common treatment, there is a critical global deficiency of donor corneal tissue (5). A global survey from 2016 revealed that approximately 53% of the worldwide population is deprived of the opportunity for corneal transplantation, with just one cornea available for every 70 required (6). Consequently, it is necessary to find a different approach for corneal reconstruction to address this issue. Regenerative medicine that involves cell culture and growth *in vitro* has emerged as a new area of research. The two main research directions are cell injection and scaffold-based corneal tissue engineering (7). Selecting the appropriate scaffold material is vital for the effectiveness of a tissue-engineered construct since its characteristics affect both the behavior of the cells developing on or inside the scaffold and the patient’s host response. For corneal tissue engineering, the scaffold materials employed must be transparent or able to achieve transparency post-implantation. Innovative approaches have been implemented to improve ocular drug accessibility by prolonging the contact duration. These encompass bio-adhesive hydrogels and gels-forming systems in situ (8). Creating colloidal particle carriers like solid noisomes, liposomes, and nanoparticles has recently gained attention (9). Researchers advanced their work by creating film-like substances to be placed in the eye, enabling the controlled release of medications over a prolonged duration. Ocular implants are solid or semi-solid film-like devices composed of medicated polymers intended for insertion into the conjunctival sac to administer medication to the ocular surface. They can function as a reservoir for controlled drug administration (10). The primary issue with these devices arises from treating these systems as foreign entities, leading to potential sensitization (11). A technique for creating these films is the solution casting or solvent casting method, which involves making a polymer solution in an appropriate solvent and then pouring it into a flat-surfaced container. Subsequently, following the solvent’s evaporation, a membrane develops at the base of the container. This approach is simple, adaptable, and does not need costly tools. It can generate a uniform and high-quality film, allowing for easy control of thickness; however, it has drawbacks like being time-intensive and leaving behind residual organic solvents (12). Ji P and colleagues, in their research, created a collagen film featuring a layered structure and high light transmission for corneal repair using a controlled solvent evaporation technique. They found that electron microscope images reveal the film produced by this approach has a dense layered structure that is similar to the microstructure of the corneal epithelial layer (13). In a different study, three methods for producing silk fibroin films were carried out through solvent casting using typical solvents, specifically aqueous solvent (aq), formic acid (FA), and hexafluoroisopropanol (HFIP), followed by three conventional techniques of post-fabrication annealing. The findings indicated that the systematic traits involve corneal cell culture under laboratory conditions, with tensile strength ranked as Aq < HF < FA. Furthermore, the findings from the annealing process indicated notable alterations in the morphotopographic, physical, degradation, and tensile characteristics. Nonetheless, the films exhibited no notable alteration in chemical composition and continued to be optically transparent, with more than 90% transmission in the visible spectrum, regardless of fabrication and post-fabrication circumstances. The movies exhibited no cytotoxicity towards L_929 _cells and were suitable for rabbit corneal cells (14).

Polyvinyl alcohol is one of the polymers that can serve as scaffolds in corneal tissue engineering. Polyvinyl alcohol (PVA) is commonly used in the biomedical sector due to its biocompatibility and lack of toxicity. Its appropriate moisture content and adjustable mechanical properties make it an excellent option for creating ocular patches (15). Notably, the biomimetic characteristics of PVA scaffolds can be enhanced by integrating natural materials(such as alginate, chitosan, and gelatin), which exhibit a greater tendency for interactions between cells and the matrix as well as between cells, allowing the 3D structure to more accurately replicate *in vivo* functions and tissue structure (16). Alongside enhancing the biological performance of PVA, creating these composite scaffolds also renders their application extremely beneficial in corneal tissue engineering because of their natural transparency (17). In a separate investigation, they looked into the creation of a new tissue-engineered corneal stroma made of bacterial cellulose (BC)/poly (vinyl alcohol) (PVA) hydrogel composite materials aimed at corneal regeneration. It was discovered that the characteristics of BC/PVA are better suited for application as corneal stromal material than BC hydrogel. Human corneal stromal cells (hCSCs) were utilized to assess the cytotoxicity of the materials, revealing that BC/PVA demonstrated remarkable biocompatibility with these cells. Additionally, *in vivo* experiments involved the intrastromal implantation of BC/PVA in rabbits. After four weeks, the cornea was almost clear, showing no significant inflammation or sensitivity (18).

Therefore, based on the explanations provided, this study aims to investigate the cell and the mechanical and eye-related properties of the films made by the solvent casting method. We used polyvinyl alcohol as the base polymer along with other polymers that have an aqueous solvent, such as alginate, gelatin, carboxymethyl chitosan, etc., which do not result in toxic residues remaining in the structure after removing the solvent. This comparative study provides strong insight into the appropriate polymer composition for corneal tissue engineering and its use in combination with drugs, plant extracts, nanoparticles, and other therapeutic agents.

## Materials and Methods

### Materials

Polyvinyl alcohol (PVA) (M_w_= 85000-124000 g mol^− 1^, hydrolysis degree: 98 ± 1%), gelatin powder (Gel) (bovine skin, type B), sodium alginate ((C_6_H_7_NaO_6_) n), medium molecular weight (216.121 g/mol), Carboxymethyl Chitosan (CMCs) (deacetylating degree 90%, molecular mass of 700 kDa, average viscosity), Carboxymethyl cellulose (CMC) (viscosity ≈ 800–1200 mPa·s, M_W_ = 2.4 × 10^4^ and the degree of carboxymethyl substitution was 0.75), Polyvinyl pyrrolidone (PVP) (M_W_ 360,000), and Polyacrilic acid (PAA) (Carbomer, high M_W_) were procured from Sigma-Aldrich (St. Louis, MO). Phosphate-buffered saline (PBS), Normal saline, and Dimethyl sulfoxide (DMSO) were obtained from Merck Chemicals (Darmstadt, Germany). Fetal Bovine Serum (FBS), Penicillin–Streptomycin (Pen-Strep), MTT ((3-(4, 5-dimethylthiazol-2-yl)-2.5-diphenyl-tetrazolium bromide), Dulbecco’s Modified Eagle Medium (DMEM), and Trypsin–EDTA were sourced from Gibco (Germany). 

### Solvent casting method

**Figure 1:**
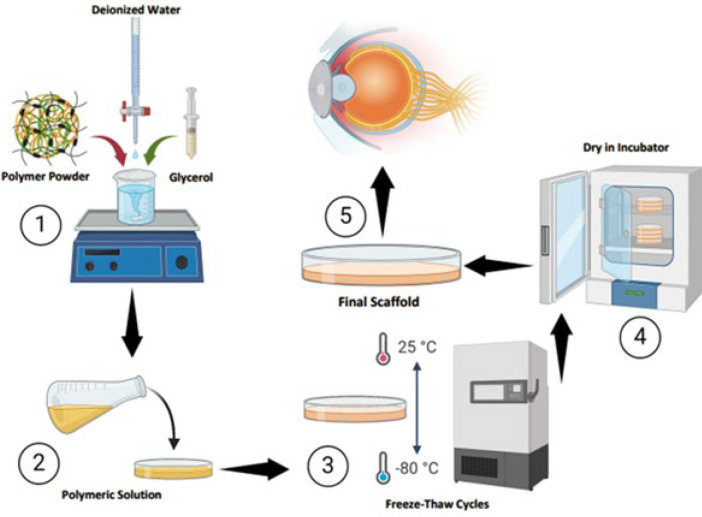
Graphical abstract of scaffold fabrication for corneal tissue engineering by the solvent casting method

At first, polymer solutions were prepared according to Table 1. Glycerol 2% by volume was added to each polymer solution and stirred. For the cross-linking process of polymers, the physical method of freeze-thaw was used in such a way that 20 hr of fast freezing at -80 °C and then slow thawing for four hours (-80 °C, -20 °C, -4 °C, and room temperature) (19). This freezing and thawing cycle was repeated 3 times for all samples. Finally, casting solutions of 15 ml of each sample were poured into 8 cm plastic Petri dishes mounted on the table of a motorized film applicator (Elcometer 4340, Elcometer, Utrecht, Belgium). The 500 μm thick wet film was dried at 40 °C and cut into rectangular strips (3 x 3 cm) (Figure 1).

### Mechanical properties

The scaffolds were subjected to mechanical testing using a uniaxial tensile testing machine with a 10 N load cell at room temperature. Cut into 40 mm and 12 mm pieces, they were subjected to a 5 kN load and extended at a 10 mm/min rate until failure. The stress-strain curve was constructed to determine the scaffold’s mechanical characteristics, including its Young’s Modulus, which was derived from the slope of the beginning segment of the stress-strain diagram (20).

### Suture retention tests

A method established by Küng et al. was used to measure the tolerance of scaffolds against suture departure (21). Rectangular films were cut with dimensions of 40 mm and 12 mm. After measuring the thickness of the compounds at the suture point, a 4-0 Vicryl suture (Polyglactin 910, Johnson and Johnson Medical) was inserted through each specimen in the inner rectangular place, maintaining a distance of 1 ± 0.2 mm from the rim. The suture’s ends were secured with a hand knot, the paper pattern was detached, and the specimen was positioned in a tensile testing apparatus (AZMA POLYMER SAM, Iran). The suture loop was secured with a bolt within the testing apparatus fitted with a 50 N load cell. The suture retention strength test was conducted at a low continuous deformation rate of 10 mm/min to ensure data reliability. The maximum force, rupture force, and deformation lengths were documented at both maximum and rupture forces. To ensure comparability of sample results, the highest force was standardized to the sample thickness.

### Water contact angle measurement

The films’ hydrophilicity was assessed using a contact angle analyzer (Sharif Solar, Model CA-500 M, Iran). To determine the static contact angle of the scaffolds, measurements were taken of a water droplet placed at different locations (22).

### Porosity assessment

To measure the porosity of the constructed scaffolds via the liquid displacement method, we used Equation 1: 

Porosity (%) = (V_1_-V_3_)/ (V_2_-V_3_) × 100 Eq. 1 

After immersing the scaffold in a starting volume (V_1_) of 96% ethanol, the volume of the ethanol becomes V_2_. After the scaffold elimination (after 1 min), the volume becomes V_3 _(23).

### Biodegradability

The degradation rate was assessed by evaluating the mass loss of the film. Three scaffold samples prepared in each group were weighed and submerged in PBS at 37 °C. After intervals of 2 hr, 6 hr, 24 hr, 48 hr, and 72 hr, the samples were taken out of PBS and placed in a 50 °C incubator until all the PBS had evaporated and the scaffolds had dried. The extent of weight reduction was evaluated using Equation 2, with W_0_ representing the beginning weight of the scaffolds and W_1_ denoting the dry weight after being taken out of water (24).

Weight loss % = (W_1_ – W_0_)/W_0_ × 100 Eq.2 

### Swelling behavior

When assessing biodegradable materials for drug delivery purposes, it has been widely accepted that swelling characteristics must be considered. The prepared films were weighed, and then a square piece (1 x 1 cm) was submerged in 3 ml of PBS at room temperature for 1 day (2, 6, and 24 hr). Samples were intermittently extracted from PBS, promptly weighed, and the quantity was computed by Equation 3 (25). M_0_ is the dried mass, and m_1 _is the swollen mass.

Equilibrium mass swelling = (m_1_ – m_0_)/m_0_ × 100 Eq. 3 

### Water vapor permeability

The water vapor permeability of the scaffolds was evaluated through a flexible bottle permeation technique. Various films were used to seal openings into which 10 milliliters of water were added. The bottles were thereafter positioned in an incubator at 37 °C. Water weight loss was measured after a period of 24, 48, and 72 hr (26). In Equation 4, ∆_W_ represents the amount of water loss, A represents the cross-sectional area of scaffolds (1.18 cm^2^), and ∆_t_ indicates the exposure time in the incubator.

WVP rate = Δ_W_/ A × Δ_t _Eq. 4

### Bacterial penetration assay

To assess the resistance of each scaffold to microbial penetration, a test was conducted using 10 ml vials, each containing 5 ml of Brain Heart Infusion (BHI) broth (Merck, Germany), which were occluded by a scaffold containing an area of 0.8 cm^2^. Cotton-filled bottles and open vials served as negative and positive controls, respectively. The test vials were stored at room temperature for 1, 3, and 7 days to observe their cloudiness as an indicator of microbial contamination. Microplate spectrophotometry readings (n=3) at 600 nm were obtained to determine the results (27).

### Hemolysis

In this experiment, 2 ml of fresh anticoagulated human blood was prepared with 2.5 ml of 0.9% sodium chloride. 200 μl of freshly diluted anticoagulated blood was incubated with circular-cut films to the size of 96-well plates at 37 °C for 60 min, followed by a 10-min centrifugation at 1500 rpm. The extracted fluid was transferred to the 96-well plate and then read at a wavelength of 545 nm (Dt). Deionized H_2_O served as a negative control (Dnc), and 0.2 ml of diluted blood in 10 ml solutions of normal saline was considered a positive control (Dpc). The extent of hemolysis was ascertained by Equation 5(28):

Hemolysis % = (D_t_ - D_nc_)/ (D_pc_ - D_nc_) × 100 Eq. 5 

### Blood clotting index (BCI)

According to previous reports, the blood clotting index is used to evaluate the coagulation properties of scaffolds. A lower BCI value indicates a higher coagulation effect. For this purpose, fresh human blood was collected in tubes containing sodium citrate and stored at 4 °C. The films (circular section with a diameter of 1 cm) were placed inside a glass beaker in a thermostatic water bath at 37 °C, and 100 μl of blood and 20 μl of 0.2 mol/l CaCl_2_ solution were added and incubated for five minutes. Then, 25 ml of distilled water was slowly added. The samples were shaken well at 37 °C, and their absorbance was determined at a wavelength of 545 nm. There was no scaffold in the control group, and the samples’ BCI was calculated using Equation 6: (29). 

Blood clotting index (%) = A _sample_ / A_ control_ × 100 Eq. 6

### pH measurement

The changes in pH values of all samples stored in normal saline (pH = 7.4) at room temperature were measured with a pH meter after 2, 4, 6, 24, and 48 hr (30).

### Transparency of scaffolds

To demonstrate the transparency of the different samples, a line of text was printed repeatedly at various font sizes, commencing at 2 pt. and culminating at 10 pt., on printer foil, utilizing Calibri as the font type. The moist samples of each category were positioned on the text to assess the legibility of the font size. UV-Vis spectroscopy was employed for quantitative transparency assessments. The samples were placed in 24-well plates and covered with 1 ml of deionized water. The transparency for each sample was determined by measuring the absorbance over the visible light spectrum (400 nm–800 nm) in 5 nm increments and turning the absorbance data into transmission data. The control group was plates filled with 1 ml of deionized water (Equation 7). For each sample type, a minimum of three measurements were taken, and the average of the spectra was computed (31).

Transparency (%) = OD _sample_ / OD_ control_ × 100 Eq. 7

### Cell viability

At first, two-way UV rays were used for 20 min to sterilize the synthesized scaffolds. The MTT assay was employed to evaluate the cytocompatibility of the prepared film. For this purpose, the human mesenchymal stem cells with a density of 1 x 10^4^ cells per circular section of each sample in the bottom of a 96-well plate in prepared media (DMEM/F12, 10% FBS, 100 units/ml of penicillin, and 100 μg per milliliter of streptomycin) was cultured in a cell culture incubator. 1-, 2-, and 3-day post-cell seeding, the culture medium was substituted with 150 μl of MTT (0.5 mg/ml), and after three hours, it was substituted with 0.1 ml DMSO. After 20 min of gentle and slow shaking of the cell suspension containing DMSO, the absorbance at wavelengths 570 and 690 nm (DMSO background wavelength) was measured in the dark condition using a microplate reader. The control comprised cells cultured on a tissue culture plate (TCP), and all tests were conducted in triplicate (32).

### Anti-inflammatory assay

The literature was consulted to evaluate the potential of preventing albumin denaturation (33). A 5 ml reaction mixture was prepared by combining 0.2 ml of 1% bovine serum albumin (BSA), 2.8 ml of PBS (pH 6.4), and 2 ml of the solution of concentrations of 5, 10, 15, 25, 50, and 100 mg/ml of polymer powder for making films. The solution was placed in an incubator set at a temperature of 37 °C for 15 min and subsequently heated to a temperature of 70 °C for five minutes. A control was established using distilled water, 0.2 ml of 1% bovine serum albumin (BSA), and 2.8 ml of PBS (pH 6.4). At last, the absorbance of the samples was measured at a wavelength of 660 nm. The reference drug, acetylsalicylic acid (ASA), was utilized at concentrations of 5 mg/ml, 10 mg/ml, 15 mg/ml, 25 mg/ml, 50 mg/ml, and 100 mg/ml. It was subjected to the same treatment and evaluated at identical absorbance levels. The % inhibition of protein denaturation was calculated using calculation 8:

Protein denaturation inhibition (%) = A _control_ – A _test_ / A _control_ × 100% Eq. 8

Variable A _control_ represents the control sample absorption, while variable A _test_ represents the test sample absorption.

### In vitro adhesiveness test

The adhesion qualities of scaffolds on ocular tissue can be replicated and assessed by adhering a wet film scaffold (in pH 7.4 buffer solution) to hand skin tissue at a movable joint with an intersection angle ranging from 0° to 120°. The adhesion of the scaffold was documented at the finger extended at 0°, flexed to 45°, 90°, and 120°, as well as when reversed and affixed to the palmar side of a finger (34).

### In vivo adhesiveness test

We used Wistar rats to investigate the adhesion of the synthesized scaffolds in the animal’s eye environment. A male Wistar rat weighing 200 to 250 g was obtained from the Royan Institute (Tehran, Iran). The animals were housed in cages on a 12-hr photoperiod while they had free access to food and water. All animal experiments were in compliance with the relevant laws, and this study was approved by the Ethics Committee of Shahroud University of Medical Sciences (registration number: IR.SHMU.REC.1403.052). First, after dividing the animals into random groups, we used Ketamine (100 mg/kg) and Xylazine (5 mg/kg) as an intraperitoneal injection to anesthetize (35). After making sure that the animals were anesthetized, we covered their corneas by cutting circularly synthesized scaffolds with a diameter of 4 mm and immersing them in Trypan Blue (0.4%) diluted with normal saline (1:1). After 10 seconds, the scaffold was brought into contact with the eye. Finally, after waking up the animals and continuing the monitoring process, the breathing rate, heart rate, and reflexes (for example, pinching the toes) were checked, and the body temperature was maintained with a heating pad. We placed rats in a warm recovery area until they were fully awake and monitored for signs of discomfort, pain, or post-anesthetic complications.

### Statistical analysis

GraphPad Prism 10.4.0.621 software and SPSS Statistics 22.0 were used to analyze all data. To compare the groups, one-factor analysis of variance (ANOVA) with Tukey’s *post* *hoc* test. Data were compared using the Kruskal-Wallis H non-parametric ANOVA test and then the Mann-Whitney U test when significant. Based on sample data, confidence interval (CI) analysis is a statistical method used to estimate the range within which a population parameter is likely to lie. Data were presented as the mean ± standard deviation (SD). The *P*-value was taken into account, and statistical significance was assigned to the value *P*<0.05.

## Results

### Mechanical tensile properties

**Figure 2:**
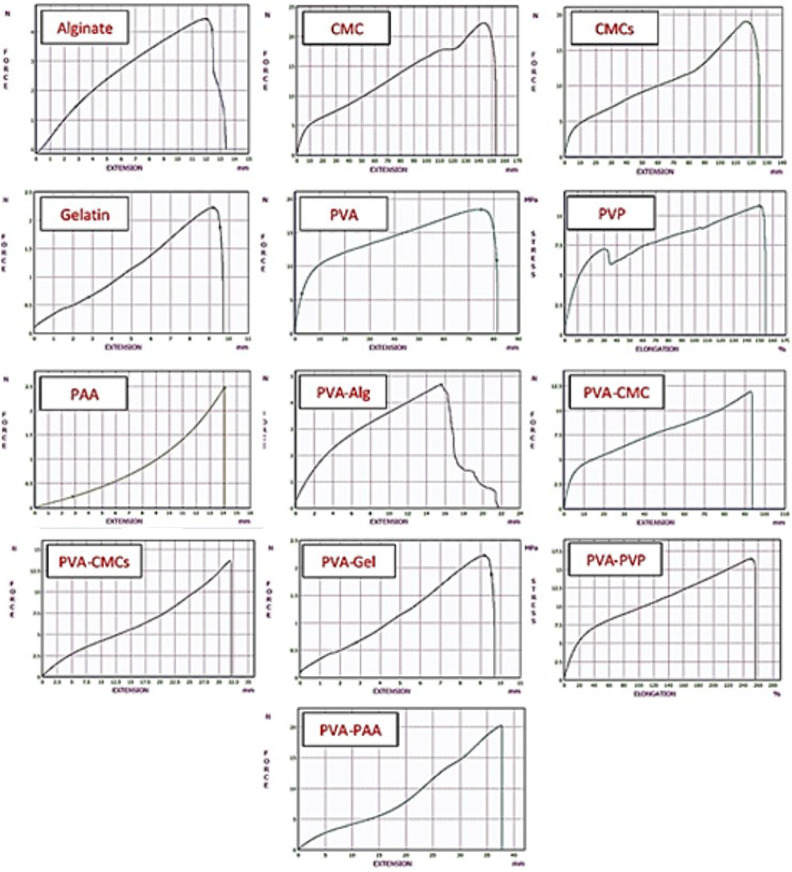
Mechanical tensile stress-strain curve of scaffolds

PVA: Polyvinyl alcohol ; Alg: Alginate; Gel: Gelatin; CMCs: Carboxymethyl Chitosan; CMC: Carboxymethyl cellulose; PAA: Polyacrilic acid; PVP: Polyvinyl pyrrolidone

Table 2 and Figure 2 present the scaffolds’ stress-strain curve and mechanical characteristics. The maximum tensile stress marks the scaffold’s initial point of failure, while elongation at break designates the elongation of scaffolds at failure points. In addition, the elastic modulus determines the slope of the linear region of the stress-strain curve. The ultimate tensile strength values ​​for PVA-PAA scaffolds are maximum, and for gelatin are minimum, which were 20.84 ± 4.55 MPa and 2.26 ± 0.09 MPa, respectively. The elastic modulus values ​​for the scaffolds were between 2 and 46 MPa, the highest value for polyvinyl pyrrolidone and the lowest for PVA-Gel were 46.49 ± 6.52 MPa and 2.12 ± 0.05 MPa, respectively. Also, confidence interval analysis was performed to compare the synthesized scaffolds with the mechanical properties of the natural human cornea based on studies (0.1 to 1 MPa elastic modulus and 3 to 7 MPa for UTS) (36, 37). PVA-CMCs and PVA-Alg samples generally resemble the mechanical tissue of the cornea more closely.

### Suture ability of scaffolds

**Figure 3:**
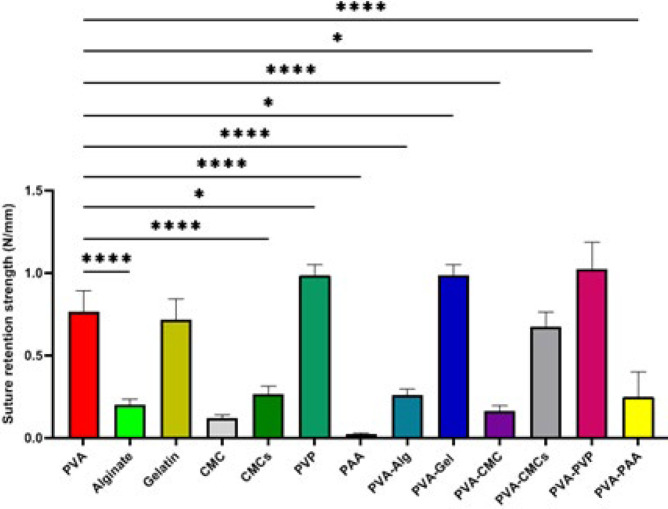
Prepared scaffolds suture ability is based on the point of rupture. all scaffolds compared to polyvinyl alcohol

Review of developed films for corneal treatment; the material’s resistance to suture release is as important as transparency. The suture ability of the samples was evaluated by quantifying each sample’s maximum tear resistance force and normalizing it to the sample thickness. The graphic in Figure 3 illustrates the suture retention strength of the fabricated films. All the synthesized compounds show suture retention strength, but PAA has lower strength than the others due to its brittleness. In addition, adding PVA to the scaffolds leads to increased suturing ability.

### Contact angle measurement

**Figure 4:**
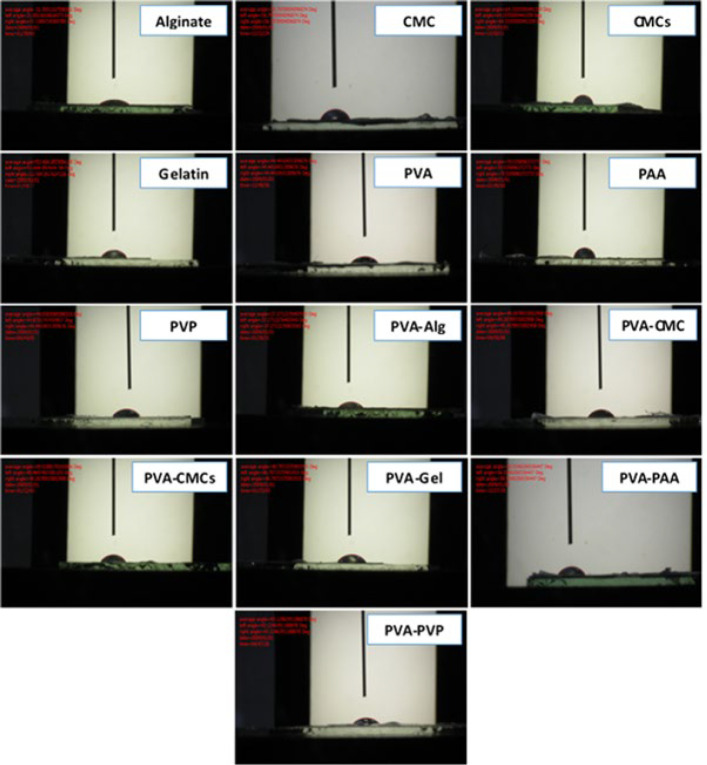
Comparison of hydrophilicity of fabricated scaffolds through contact angle is 10 seconds

PVA: Polyvinyl alcohol ; Alg: Alginate; Gel: Gelatin; CMCs: Carboxymethyl Chitosan; CMC: Carboxymethyl cellulose; PAA: Polyacrilic acid; PVP: Polyvinyl pyrrolidone

The scaffold wettability was investigated through the performance of the water contact angle test. Surfaces that possess hydrophilic properties are commonly acknowledged to have a contact angle below 90°, while hydrophobic surfaces have a contact angle over 90° (38). Moreover, lower contact angles correspond to greater wettability. In the corneal environment, it has been shown that, like the tear process, hydrophilicity facilitates superior cell adhesion and proliferation. Table and Figure 4 show the values ​​of the minimal water contact angle for alginate and the maximum average angle for PAA, which were measured at 31.09 ± 2.51° and 70.53 ± 4.66°, respectively. The water contact angles of less than 90° suggest that all scaffolds have a hydrophilic character, according to the findings. Furthermore, based on previous studies, the contact angle of the human cornea varies from 30 to 40° (39). With confidence interval (CI) analysis, it can be concluded that the alginate and PVA-Alg samples are more similar to the natural corneal tissue.

### Porosity

**Figure 5:**
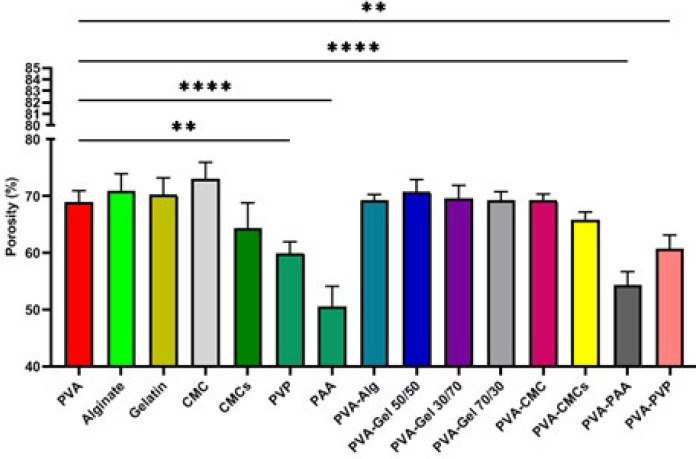
Porosity histogram of scaffolds compared to polyvinyl alcohol

Based on the liquid displacement method, the porosity of different groups was evaluated, and the results are presented in Figure 5. Porosity values ​​show statistically significant differences between different scaffolds. The lowest amount of porosity was related to the PAA scaffold, and the highest was associated with CMC, with values ​​of 50.66 ± 5.19 and 73.01 ± 4.27 %, respectively.

### Swelling behavior

**Figure 6:**
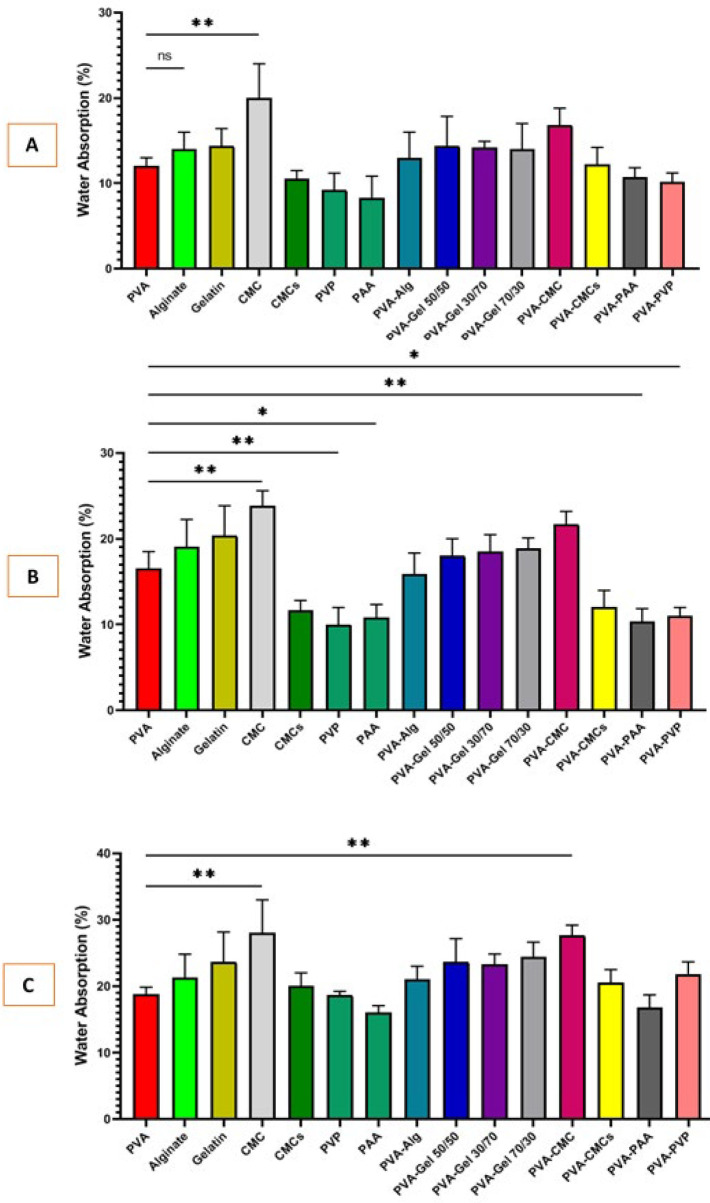
Potential for water absorption of scaffolds after 2H 6H, and 24H,inubation in 37C and PBS compared to polyvinyl alcohol

Figure 6A-C shows the swelling behavior of different polymer scaffolds. The interaction between polymer chains and water molecules can cause swelling. This swelling behavior of the scaffold provided a suitable three-dimensional structure that is the native microenvironment of the cells, thereby promoting cell survival, migration, and proliferation. The findings showed that the highest swelling was for the CMC scaffold and the lowest was for the PAA scaffold, which was 28.13 ± 6.75 and 16.06 ± 2.33%, respectively, at 24 hr after incubation.

### Weight loss analysis

**Figure 7:**
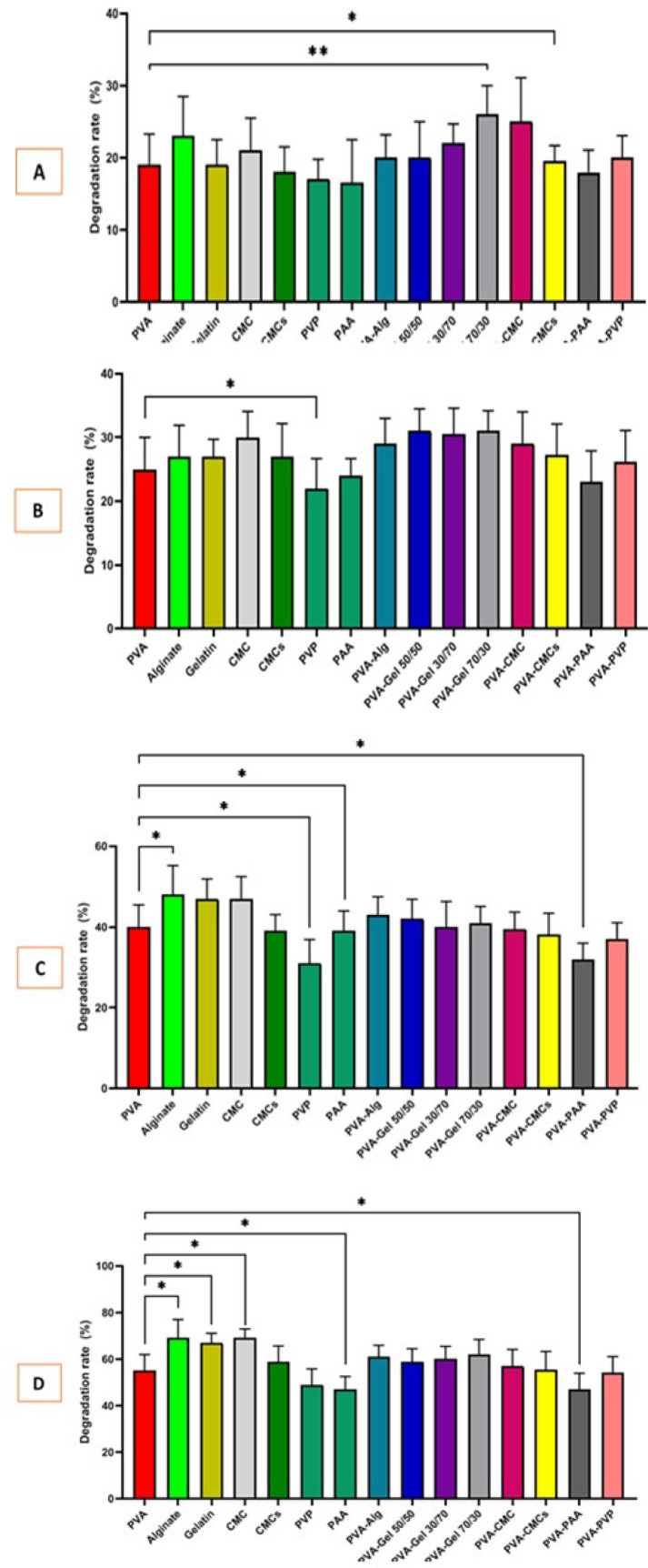
Degradation histogram of scaffolds after 2H 6H, 24H, 48H, and 72H inubation in 37C and PBS compared to Polyvinyl alcohol

The scaffold degradation rate should correspond to the rate of regeneration and repair of the target tissue. A lower speed can impair the replacement of the scaffold with formed tissue, while a higher speed can lead to an incomplete healing process. The weight loss results of the prepared scaffolds are described in Figure 7A-E. It can be concluded that the highest weight loss is related to alginate and the lowest degradation is associated with PAA, with values ​​of 80.43 ± 9.65% and 58.03 ± 7.39% after 72 hr of incubation in a PBS environment.

### Water vapor permeability capacity

**Figure 8:**
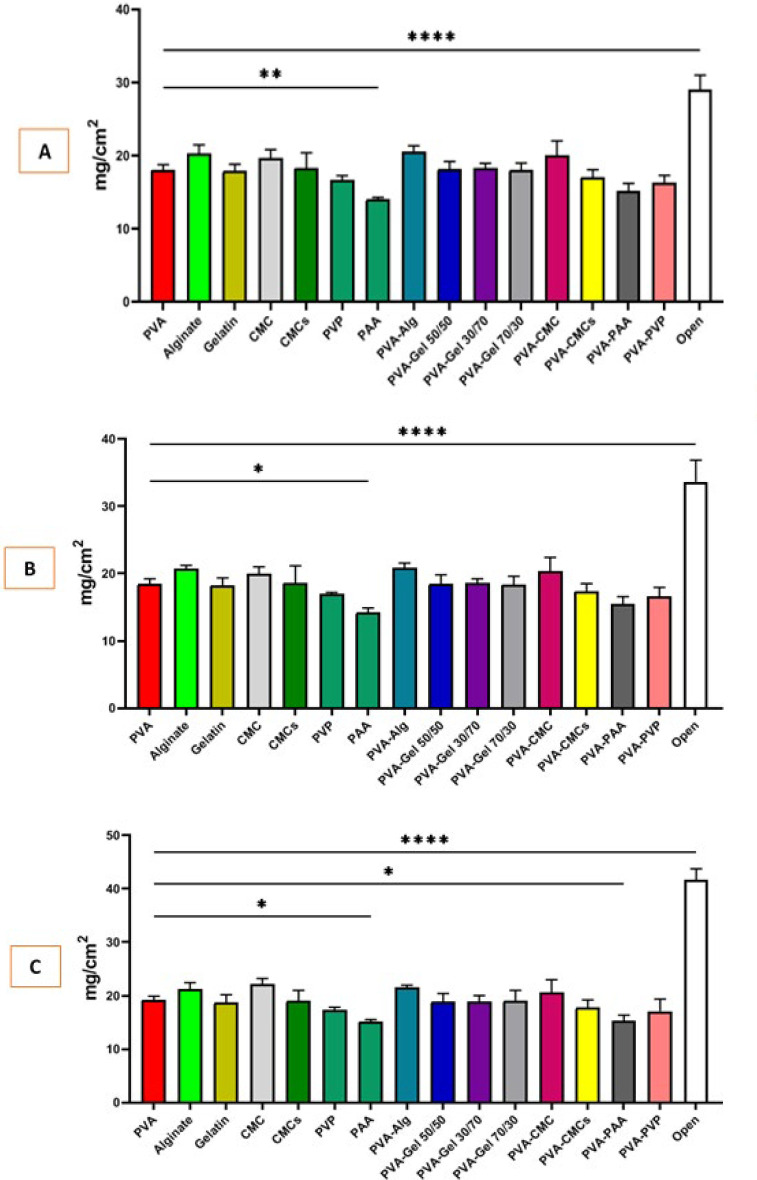
(A) Water vapor permeability of scaffolds after 24 hr incubation, (B) Water Vapor Permeability of scaffolds after 48 hr incubation, and (C) water vapor permeability of scaffolds after 72 hr incubation

Effective dressings must be able to regulate gas transport through their structure to improve the healing process. High water vapor permeability (WVP) can accelerate the drying of the damaged area and lead to scar tissue formation. Conversely, exudate can accumulate when WVP levels are low, leading to delayed healing and increased susceptibility to infection (40). As shown in Figure 8A-C, our findings show that the group treated with CMC scaffold showed the highest level of water vapor permeability (22.16 ± 2.39 mg/cm^2^), and PAA showed the lowest with 15.21 ± 1.66 mg/cm^2^.

### Bacterial penetration test

**Figure 9:**
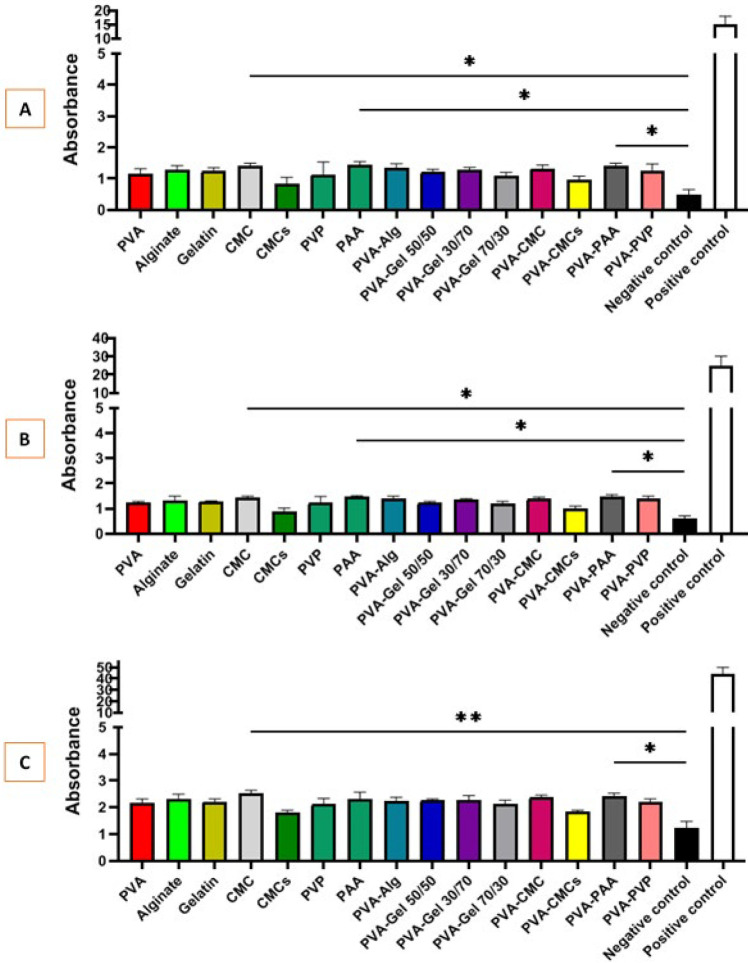
Bacterial penetration through prepared scaffold after 1, 3 , and 7 days to SBF compared to negative control

The leading cause of delayed healing of wounds, including corneal wounds, is bacterial infection, so the vital role of antibacterial activity in scaffolds during the healing process is emphasized. Figure 9A-C shows the consequences of bacterial penetration through the synthesized scaffolds. The investigation showed that the test tubes of BHI broth covered with films containing carboxymethyl chitosan (CMCs) showed less bacterial growth compared to others after seven days, which did not exhibit a statistically significant change relative to the negative control group. The positive control group exhibited a significantly darker liquid color in BHI broth than all other groups.

### Blood compatibility analysis

**Figure 10:**
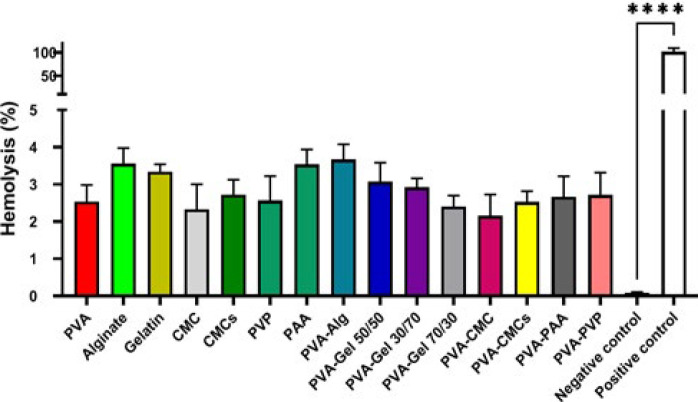
Anti-coagulated human Blood compatibility of prepared scaffold compared to negative control

The compatibility of the scaffolds with blood cells, particularly erythrocytes, is necessary to ensure that the repair process is successful. The interaction between the implanted scaffold and erythrocytes is a primary event that triggers inflammatory responses (41). To assess compatibility, synthesized films were tested with erythrocytes taken from a healthy volunteer, and the amount of liberated hemoglobin was determined with a microplate reader. The results of Figure 10 show that the hemolysis caused by the treatment with all scaffolds is less than that of the positive control, which shows that the prepared scaffolds are blood compatible and have less than 4% hemolysis. However, the scaffold containing PVA-Alg had the highest and PVA-CMC the lowest amount of hemolysis, with values ​​of 3.65 ± 0.29% and 2.15 ± 0.41%, which are not statistically markedly distinct from the negative control group. 

### Blood clotting index

**Figure 11:**
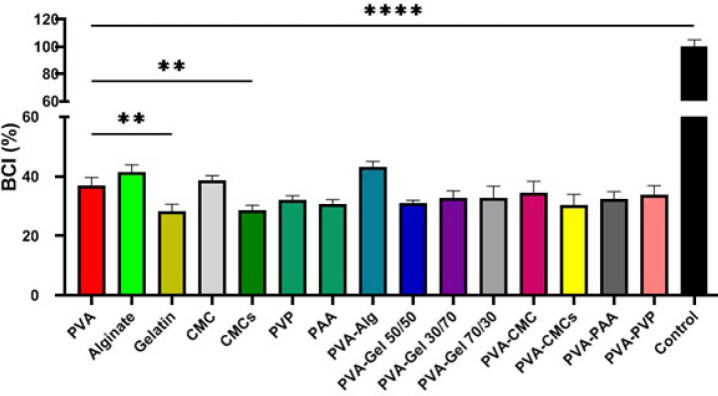
Blood clotting analysis of prepared scaffolds by fresh human blood compared to polyvinyl alcohol

A study was conducted using the blood clotting index (BCI) to assess the anti-thrombogenic characteristics of a material in human blood. The BCI value is generally inversely related to the coagulation effect of the material, with lower values indicating better anti-thrombogenicity (42). Human blood droplets were applied to scaffolds to evaluate clot formation, and absorbance measurements were taken after 60 min to determine the amount of free hemoglobin released from clotted blood. As a result, in Figure 11, the absorbance values ​​were converted to the percentage of free hemoglobin and calculated as BCI values ​​for each sample. PVA-Alg scaffold recorded the highest blood clotting index with 41.33 ± 2.97%, and the gelatin scaffold the lowest with 28.21 ± 3.09%. Therefore, the PVA-Alg scaffold has stronger anticoagulant activity, and gelatin has lower activity.

### pH measurement

Various scaffold compositions’ pH levels were assessed using a digital pH meter. This experiment was performed in three repetitions. For this purpose, the pH was measured over time in 0, 2, 4, 6, 24, 48, and 72 hr and reported in Table 4. All scaffolds had a pH between 6 and 8, the most acidic of which was PAA with 5.31 ± 0.03 and the most alkaline CMCs with 7.69 ± 0.12. 

### Optical properties

**Figure 12:**
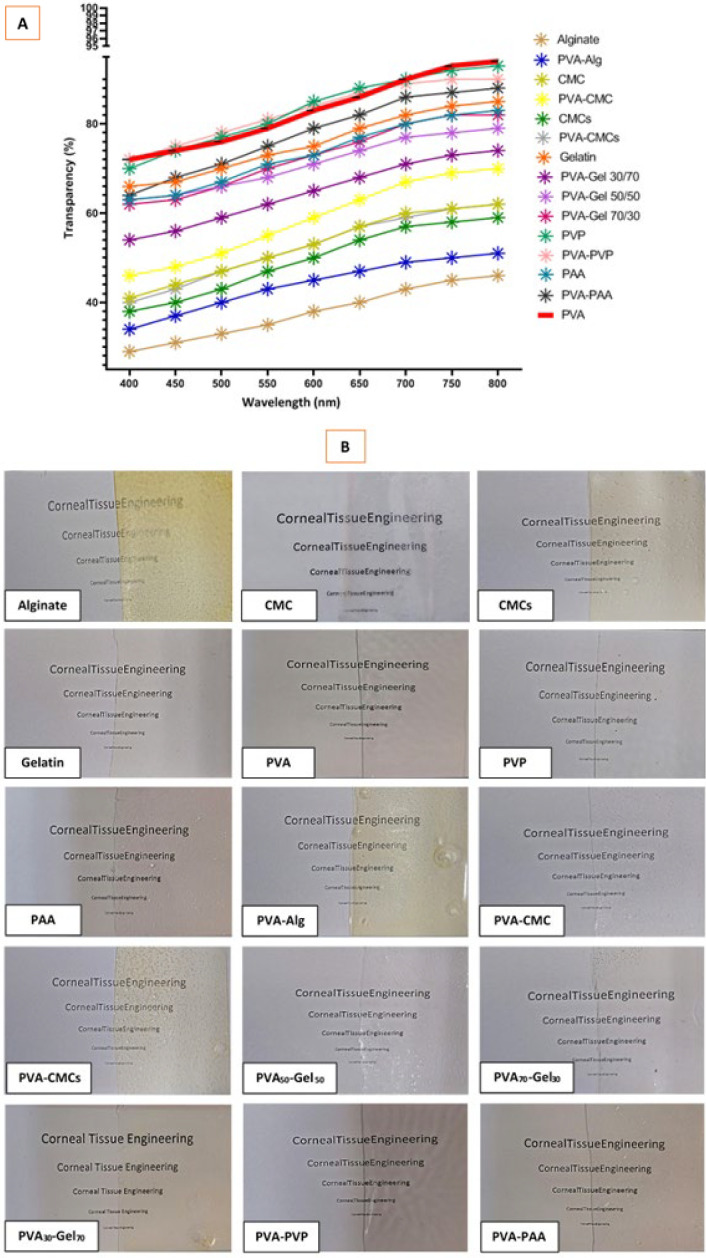
(A) Outcomes of the UV-VIS transparency assessment of fabricated scaffolds. (B) Representative images of scaffolds in transparency analysis utilizing the legible font size approach as references for plain text

The transparency of the scaffolds is essential for corneal application; therefore, we employed two approaches to test the transparency of our compounds. Figure 12 summarizes the transparency spectra of the compounds within the visible light spectrum, spanning 400 to 800 nm, as measured in triplicate using a UV-Vis spectrophotometer for each sample. The spectra for the fabricated scaffolds are shown as lines. Polyvinyl alcohol (PVA) was measured as a reference substance and is shown in the diagram as a solid red line. The transparency spectrum for all samples shows a curved shape with low transparency at a small wavelength and increasing transparency with increasing wavelength. As the most important parameter, transparency at 400 nm was used for further comparisons. A significant difference can be seen among the composites based on Figure 11A. Composites containing PVP-PVA show more than 70% translucency at 400 nm, while samples containing alginate lead to translucency of about 29% at 400 nm. Based on the macroscopic images, it is possible to see the transparency of the synthesized samples, which is consistent with their quantitative analysis (Figure 12B). Based on the general results of this test, at 800 nm, alginate has the lowest optical transmittance (about 46%), and PVA has the highest optical transmittance (about 93%) and, by nature, the highest level of transparency.

### Cell viability assessment

**Figure 13:**
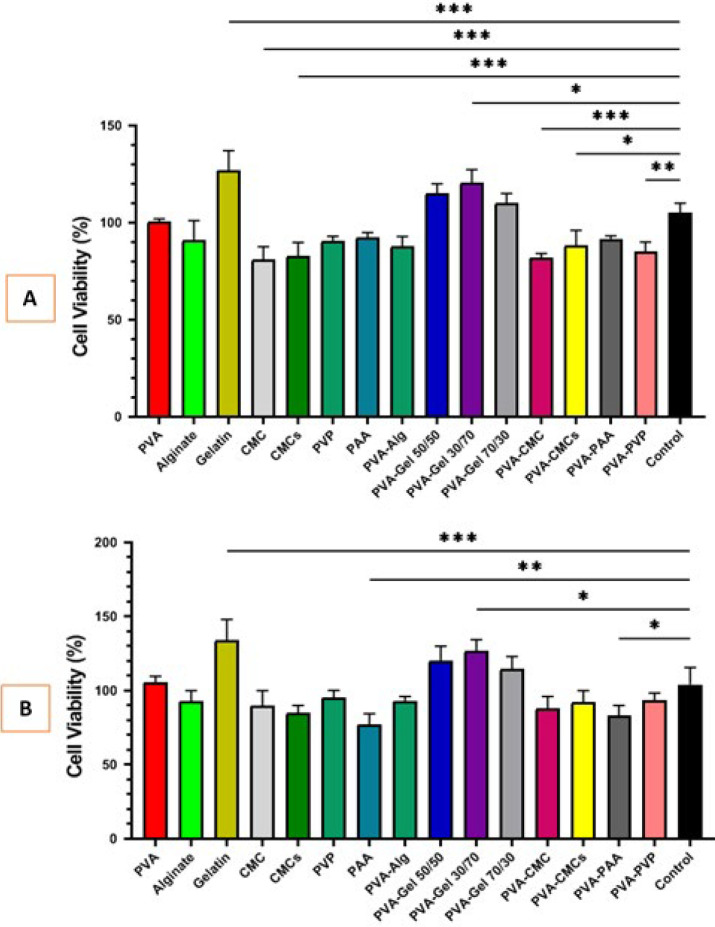
(A) Cell viability histogram for human mesenchymal stem cells that have been measured with MTT assay in prepared scaffolds after 24 hr incubation, (B) Cell viability histogram for human mesenchymal stem cells that have been measured with MTT assay in prepared scaffolds after 72 hr incubation

MTT assay was performed at 24 and 72 hr to further evaluate the effect and biocompatibility of the synthesized scaffolds on the activity and viability of human mesenchymal stem cells, as shown in Figure 13. 24 hr after treatment and direct contact of scaffolds with human mesenchymal stem cells, all studied groups showed good cell compatibility, CMC showed the lowest value with 82 ± 7.92%, and gelatin showed the highest cell viability with 127 ± 11.38% (Figure 12A). After 3 days of treatment, the scaffolds did not show significant cytotoxicity. All treatment groups survived above 75%, thus making them suitable for direct contact with corneal cells *in vivo*. Finally, PAA was the lowest and gelatin was the highest with 77.33 ± 3.13 % and 134 ± 12.45%, respectively. In addition, only PAA and PVA-PAA treatment groups were associated with decreased cell viability after 72 hr (Figure 13B).

### Anti-inflammatory test

**Figure 14:**
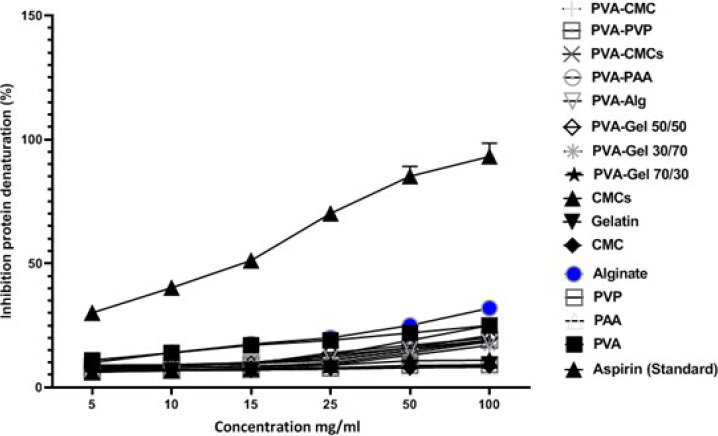
The study investigated the ability of various concentrations (5, 10, 15, 25, 50, and 100 mg/ml) of different polymers to prevent the denaturation of proteins caused by heat, in comparison to acetylsalicylic acid (Aspirin) which was used as a reference medicine

The protein denaturation process was employed to assess the anti-inflammatory characteristics of the substance. This experiment aimed to evaluate the anti-inflammatory effects *in vitro* of different polymers in synthesizing scaffolds, unlike acetylsalicylic acid, a common standard anti-inflammatory agent. Analysis was performed by measuring the degree of denaturation of BSA, or bovine serum albumin (Figure 14). PVA, CMC, and PVA-CMC scaffolds showed more inhibitory effects than other treatment groups. The highest inhibition levels of BSA denaturation by alginate at a concentration of 100 mg/ml resulted in an inhibition percentage of 31.33 ± 2.97%. The common drug acetylsalicylic acid showed inhibitions of 32.06 ± 1.29% and 90.18 ± 3.42% in concentrations of 5 and 100 mg/ml, respectively.

### Adhesion properties

**Figure 15:**
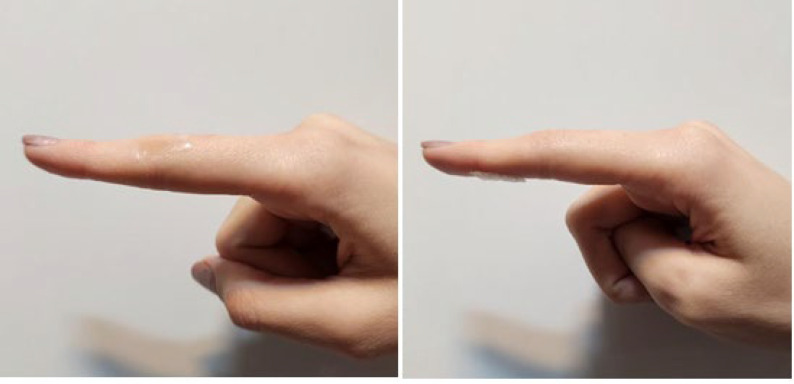
Comparison of bio-adhesion of prepared scaffolds at different angles of the human hand ( at 0◦ and the back of the finger (palm side)

**Figure 16:**
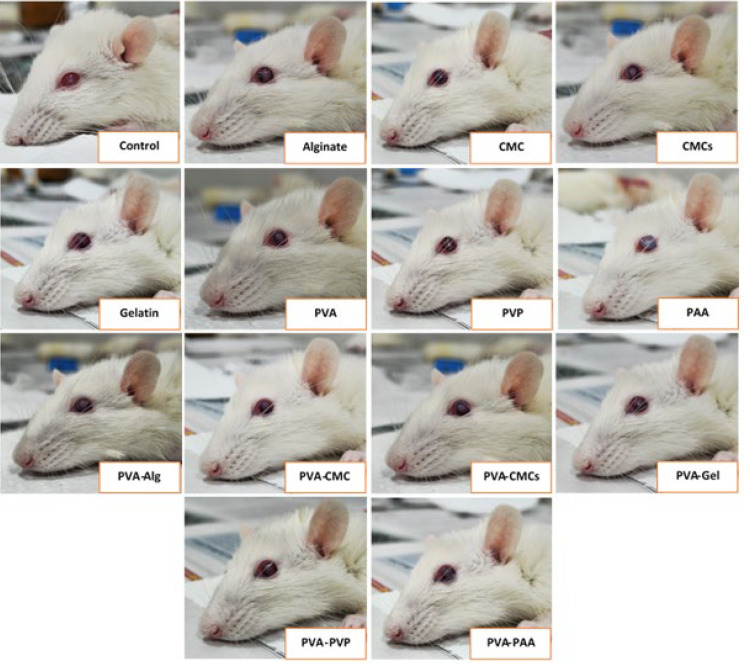
Demonstration of the adhesion of the synthesized scaffolds on the corneal arch of rats and taking a photo after ten seconds

Various scientific methodologies have been devised to enhance the tissue adhesion characteristics of scaffolds, including the augmentation of surface forces with tissues. To facilitate the simulation of the adhesive properties of casted scaffolds to the surface of the eye, it was tested by connecting the scaffold to the skin tissue on a movable joint, and the displayed images are presented in Figure 15. As evidenced by Table 5, stable fixation of the scaffold on the tissue was achieved with an intersection angle from 0◦ to 120◦ (0◦, 45◦, 90◦, 120◦, and palm side). Based on the results listed in the table, PVA, CMC, gelatin scaffolds, and their combination had adhesion in all the investigated angles. After placing the films stained with Trypan blue color and placing them on the corneal arch of the rat, after ten seconds, photography was done (Figure 16). Based on the recorded images, it can be seen that the synthesized PVA, CMC, and gelatin films are well fixed in place, and no slipping was observed. However, the PAA films appeared disappointing, indicating their complete inability to adhere to the moist and mucosal surfaces of the eye.

## Discussion

Developing biomaterials for corneal tissue engineering requires a delicate balance between cellular compatibility, mechanical integrity, and optical transparency. This study systematically compared these critical properties across a range of natural and synthetic polymers, including polyvinyl alcohol, alginate, gelatin, carboxymethyl chitosan, carboxymethyl cellulose, polyacrylic acid, and polyvinyl pyrrolidone, to identify optimal candidates for corneal regeneration.

In the solvent casting technique, a polymer is dissolved in a solvent, and the mixture is poured into a mold (43). Different methods, like air drying, vacuum drying, and freeze drying, can remove the solvent. The surface properties of the films depend on the solvent used. We chose the solvent casting method for making corneal scaffolds because it is effective for creating thin films, as shown in Table 6. Although electrospinning and 3D bio-printing are alternatives, limitations in polymer selection and costs led us to avoid them (44). To address the solvent toxicity, we used biocompatible polymers soluble in water and evaluated the properties of the films produced.

The modulus of elasticity, also known as the elastic modulus, measures a material’s resilience and resistance to temporary deformation (45). This concept is also related to the material’s ability for suturability because it means the ultimate strength tolerance before tearing. The study finds that the elastic modulus of PAA, CMCs, gelatin, and PVA-Gel films is near the standard range. Scaffolds with alginate and PVA-Alg meet UTS standards, while gelatin, PVA-Gel, and PAA samples are also close. Despite standard elastic modulus and UTS, PAA is not recommended due to its fragility and low flexibility. However, considering both the elastic modulus and UTS, the PVA-CMCs and PVA-Alg samples overall resemble the mechanical tissue of the cornea more closely. The suturability of engineered scaffolds is crucial for the cornea, as it helps secure wound dressings on its surface. Suturable dressings provide better mechanical support during blinking and eye movements (46). PVA, gelatin, PVP, PVA-Gel, and PVA-PVP scaffolds are ideal because of their elasticity and strength. 

In corneal tissue engineering, the hydrophilicity of scaffolds is crucial, as the natural cornea is mostly water. A hydrophilic surface improves cell connection and growth, mimicking the corneal environment and enhancing nutrient and oxygen exchange, while also reducing non-specific protein absorption and inflammation (14). Materials with water contact angles of 0 to 90° are considered hydrophilic, and all samples showed average angles below 90° due to the polymers’ hydrophilic nature (47).

High porosity in scaffolds designed for corneal tissue creation is essential for facilitating cell migration, adhesion, differentiation, proliferation, and optical clarity. Based on the results of past studies, the percentage of porosity between 50 and 80 percent and the pore size of 10 to 100 µm are suitable for corneal wound dressings (40). A porosity percentage greater than 50% leads to greater mechanical strength and, to some extent, improves transparency. On the other hand, a porosity of more than 80% supports the permeability of liquids, oxygen, and nutrients. 

Recent investigations have revealed polymers that create scaffolds with hydrophilic or hydrophobic functional groups and can swell when absorbing water (40). This increases pore size, allowing for better cell attachment and growth. Also, Moisture reduces friction and irritation in the wound area, leading to less discomfort for the patient. Hydrated scaffolds speed up healing and decrease irritation on the eye surface (48). In addition to water absorption, biodegradability is essential as it reduces the need for dressing changes and their associated irritation. This is linked to the swelling ability and porosity of the scaffolds. All scaffolds in this study are hydrophilic, with more porous samples absorbing more water, which can lead to degradation. Proper biodegradability allows for the slow release of components to avoid sudden accumulation at the repair site (40). Our study’s investigation in PBS medium is incomplete, but it yielded significant results relating water absorption and biodegradability to porosity. Samples containing CMC, alginate, and gelatin (49) showed the highest absorption and degradation due to their hydrophilic nature. Other polymers also showed promising results after incubation. Although hydrophilic, the PAA (50) polymer has lower swelling and biodegradability, probably due to its lower porosity. Water vapor permeability is a key factor in healing corneal and eye scars. It helps balance fluid and moisture on the eye, delivers oxygen, and lowers infection risks (40). Carboxymethyl cellulose had higher permeability due to its hydrophilic structure and porosity, while PAA had lower permeability because of its low porosity.

Bacteria like Corynebacterium diphtheriae, Haemophilus influenzae, Neisseria gonorrhoeae, Neisseria meningitidis, and Listeria species can penetrate the corneal epithelium. Bacterial infections delay corneal wound healing, highlighting the need for antibacterial activity in scaffolds (51). A bacterial penetration test showed no significant differences between treatment groups except for carboxymethyl chitosan. Based on previous knowledge about the antibacterial properties of CMCs, it can be said that this is due to the presence of positively charged amino groups in CMC, which establish electrostatic bonds with negatively charged components present in the bacterial cell membrane (such as lipopolysaccharides and teichoic acids) (52).

Sarika *et al*. indicated that the extent of hemolysis is affected by the compatibility of certain materials with blood. The hemoglobin concentration in the supernatant correlates directly with the extent of red blood cell destruction. Previous investigations indicate that the crucial threshold for hemolysis is below 5% (53). Therefore, due to the use of polymers whose biocompatibility has already been proven, all samples are within the standard range of biomaterial hemolysis, and no particular sample is favored over the others.

The assessment of coagulation activity plays a significant role in evaluating and optimizing biomaterials used in corneal repair. The lower the BCI value, the more effective the coagulation effect of the dressing (54). The cornea’s lack of blood vessels is essential because uncontrolled bleeding can cause infection and inflammation. Blood coagulation creates a fibrin network that helps stabilize wounds. Too much or too little blood can harm corneal healing. Chitosan and its derivatives speed up clot formation by interacting with blood cells, while gelatin enhances coagulation by absorbing blood and forming clots (55). On the other hand, alginates, especially when they are sulfated, can have anticoagulant effects by reducing platelet adhesion and activity (56). Our study results align with existing knowledge. Scaffolds made of gelatin and chitosan showed a lower BCI, which is helpful for surgeons in post-operative dressings. Alginate scaffolds are known for their anticoagulant properties, increased BCI, lowered neovascularization risk, and prevention of thrombosis.

PH adjustment is an effective factor for the compatibility of a biomaterial and the success of its performance in the process of implant and restoration. In addition, chemical eye injury is an ophthalmic emergency, which may be caused by exposure to an acidic (pH<4) or an alkali (pH>10) solution to the eye (57). According to studies (58), the human eye’s pH is neutral to slightly alkaline, but it is mainly in the range of 7 to 7.4. 

Corneal regeneration involves many types of corneal tissue cells, but the regulatory framework is poorly understood. It is crucial to balance the growth and replacement of corneal epithelial cells to keep the cornea clear and protect the eye (59). Providing a suitable environment for epithelial cells is essential when using biomaterials as corneal wound dressings. Gelatin, a biopolymer from collagen, mimics the extracellular matrix, helping cells attach, interact, proliferate, and differentiate. Gelatin has RGD domains that facilitate cell binding, promoting more cell growth than other polymers (60). This is also true for PVA, PAA, and PVP because they do not provide a suitable cell attachment site for growth and proliferation, especially PVP, which has a low propensity to interact with cell surface receptors and absorb proteins. In addition to not having bioactive sites, carboxymethyl cellulose also limits cell attachment due to providing very hydrophilic surfaces (61). Finally, due to the use of polymers in this study, whose compatibility has already been proven and the results of favorable cell evaluation (above 75% viability), it is possible to improve cell adhesion and adhesion by biological and chemical modifications such as polymer surface coating. 

The leading causes of infectious keratitis include infections, exposure to certain eye drugs, excessive UV radiation, welding, and intense sunlight (62). High levels of inflammation from cytokines like TNFα and IL_1_α damage eye cells and their structure. Chronic inflammation can lead to excessive collagen _III_ production and more fibroblasts, causing scarring and loss of corneal clarity (63). This highlights the need for anti-inflammatory materials for the cornea. Although known anti-inflammatory compounds weren’t used in this study, a group with more alginate showed better results, likely due to its anti-oxidant properties.

The cornea is the eye’s outer layer that needs to stay clear for light to reach the retina. Using an opaque material for corneal healing can cause vision loss until the material breaks (64). Sound light transmission is essential for the material in corneal repair. The human cornea detects visible light in the range of about 400 to 800 nm through cone cells in the retina (65). The evaluation of transparency and light transmittance in constructed scaffolds is crucial. Transparency levels vary by material; hydrogels are more transparent, while electrospun scaffolds are less so. Studies show an ideal cornea transmission rate of about 90% (66). In this study, polymers with good light transmittance were used. PVA samples have a transparency of about 93%, making them desirable for use. The direct carbon chains and hydroxyl groups in PVA help prevent light scattering and ensure sound light transmission. Its refractive index is similar to visible light, which reduces reflection and scattering. Therefore, PVA is recommended to create a transparent scaffold. Conversely, alginate is less effective in light transmittance and transparency due to impurities, natural pigments, and mostly amorphous structures (16).

Traditional eye dressings have drawbacks, such as needing sutures or other fasteners that don’t ensure proper wound drainage and lower healing effectiveness. Synthesized scaffolds are essential for corneal wound healing due to their strong adhesion (67). Due to the interaction with the tear layer of the cornea and the presence of functional groups (-COO-, OH-, and NH_2_-), the hydrophilicity capacity contributes to their adhesive properties by increasing the establishment of hydrogen bonds and electrostatic interactions between biological materials and eye tissue. Carboxymethyl cellulose has good adhesive properties due to its high ability to absorb liquids and form hydrogen bonds with surrounding molecules through hydroxyl (OH-) and carboxylate (-COO-) groups and its higher molecular weight. Gelatin can facilitate tissue adhesion owing to a large number of hydrogen bond-forming sites such as carboxyl (COOH-), amine (NH_2_-), and hydroxyl (OH-) groups. Polyvinyl alcohol also creates a strong hydrogen bond with hydrophilic surfaces due to having many hydroxyl (OH-) groups (68). In this study, CMC, gelatin, and PVA scaffolds had the highest adhesion. Therefore, it is hoped that they will be used and not slip off the ocular surface.

According to the findings of this study, a more in-depth approach is obtained for selecting polymers for corneal tissue engineering. We cannot choose the best sample for this study because each polymer has properties that make it suitable for its application. For example, if maximum clarity is desired, PVA, and if cell compatibility is of maximum importance, gelatin is the optimal choice. Therefore, we leave it up to the reader to choose the sample according to the purpose of the study. One of the most critical limitations of comparative biological studies at this level is the batch-to-batch variability because the polymers studied vary greatly in origin, composition, molecular weight, and quality, which can lead to different results. Also, to check the properties of biodegradability and liquid absorption, it is better to use the aqueous environment of the human eye (aqueous humor) for a better and more accurate simulation. There are also other polymers, such as PLGA, PCL, PCGA, PEG-PLGA, hyaluronic acid, collagen, etc., that can be evaluated for this purpose. Therefore, it is recommended that they be considered and characterized comparatively in future studies.

**Table 1:** Formulation of polymer solutions prepared for film preparation using the solvent casting method

***Polymer***	**Ratio**	**Concentration**	**Preparations information**
*Polyvinyl alcohol *	100	3%	Water soluble, temperature 70 °C, stirring 24 hr at 500 rpm
*Alginate *	100	2%	Water soluble, room temperature, stirring 24 hr at 500 rpm
*Gelatin *	100	3%	Water soluble, temperature 40 °C, stirring 8 hr at 450 rpm
*Carboxymethyl Chitosan *	100	3%	Water soluble, room temperature, stirring 8 hr at 500 rpm
*Carboxymethyl cellulose *	100	2%	Water soluble, room temperature, stirring 24 hr at 350 rpm
*Polyacrilic acid *	100	2%	Water soluble, room temperature, stirring 8 hr at 500 rpm
*Polyvinyl pyrrolidone *	100	4%	Water soluble, temperature 40 °C, stirring 24 hr at 400 rpm
*PVA-Alg*	50/50	3%-2%	Water soluble, room temperature, stirring 24 hr at 450 rpm
*PVA-Gel*	30/70	3%-3%	Water soluble, room temperature, stirring 8 hr at 500 rpm
*PVA-Gel*	50/50	3%-3%	Water soluble, room temperature, stirring 8 hr at 500 rpm
*PVA-Gel*	70/30	3%-3%	Water soluble, room temperature, stirring 8 hr at 500 rpm
*PVA-CMCs*	50/50	3%-3%	Water soluble, room temperature, stirring 8 hr at 500 rpm
*PVA-CMC*	50/50	3%-2%	Water soluble, room temperature, stirring 24 hr at 350 rpm
*PVA-PAA*	50/50	3%-2%	Water soluble, room temperature, stirring 8 hr at 500 rpm
*PVA-PVP*	50/50	3%-4%	Water soluble, room temperature, stirring 8 hr at 400 rpm

PVA: Polyvinyl alcohol ; Alg: Alginate; Gel: Gelatin; CMCs: Carboxymethyl Chitosan; CMC: Carboxymethyl cellulose; PAA: Polyacrilic acid; PVP: Polyvinyl pyrrolidone

**Table 2:** Mechanical properties of scaffolds, statistical comparison with PVA scaffold, and confidence interval comparison with human corneal tissue

***Sample***	**Ultimate Tensile Stress (MPa) **	**Confidence Interval (CI)**	**Is there a significant difference?**	**Elastic module ** **(MPa)**	**Confidence Interval (CI)**	**Is there a significant difference?**
*Polyvinyl alcohol *	18.01 ± 2.71	11.27 to 24.7	NO	32.96 ± 3.77	23.5 to 42.3	YES
*Alginate *	4.31 ± 0.96, *P* <0.0001	1.92 to 6.7	YES	10.58 ± 2.28, *P* <0.0001	4.9 to 16.2	NO
*Carboxymethyl cellulose *	22.45 ± 3.16, *P* = 0.0086	14.6 to 30.2	YES	3.40 ± 0.62, *P* <0.0001	1.8 to 4.9	NO
*Carboxymethyl Chitosan *	17.95 ± 2.35, P>0.9999	12.1 to 23.7	YES	2.57 ± 0.39, *P* <0.0001	1.6 to 3.5	NO
*Gelatin *	2.26 ± 0.09, *P* <0.0001	2.03 to 2.4	YES	2.41 ± 0.25, *P* <0.0001	1.7 to 3.03	NO
*Polyvinyl pyrrolidone *	12.33 ± 1.04, *P* = 0.0012	9.7 to 14.9	YES	46.49 ± 6.52, *P* <0.0001	30.2 to 62.6	YES
*Polyacrilic acid *	2.46 ± 0.06, *P* <0.0001	2.3 to 2.6	YES	0.34 ± 0.08, *P* <0.0001	0.14 to 0.53	YES
*PVA-Alg*	4.61 ± 0.19, *P* <0.0001	4.13 to 5.08	NO	11.23 ± 1.89, *P* <0.0001	6.5 to 15.9	NO
*PVA-CMC*	11.92 ± 1.57, *P* = 0.0013	8.01 to 15.8	YES	7.99 ± 1.51, P <0.0001	4.23 to 11.7	NO
*PVA-CMCs*	13.55 ± 3.12, *P* = 0.0215	5.7 to 21.3	NO	4.95 ± 1.27, *P* <0.0001	1.7 to 8.1	NO
*PVA-Gel*	2.29 ± 0.14, *P* <0.0001	1.9 to 2.6	YES	2.12 ± 0.05, *P* <0.0001	1.9 to 2.2	YES
*PVA-PVP*	16.16 ± 2.86, *P* = 0.8576	9.05 to 23.2	YES	21.81 ± 4.67, *P* <0.0001	10.2 to 33.4	YES
*PVA-PAA*	20.84 ± 4.55, *P* = 0.2957	9.5 to 32.1	YES	4.06 ± 0.99, *P* <0.0001	1.6 to 6.5	NO

The values are presented as the mean ± SD, with a sample size of n = 3. ns = *P*˃0.05, *P<0.05, **P<0.01, ***P<0.001, and ****P<0.0001.

PVA: Polyvinyl alcohol ; Alg: Alginate; Gel: Gelatin; CMCs: Carboxymethyl Chitosan; CMC: Carboxymethyl cellulose; PAA: Polyacrilic acid; PVP: Polyvinyl pyrrolidone

Values represent the mean ± SD, n = 3, *P<0.05, and ****P<0.0001. SD: standard deviation.

PVA: Polyvinyl alcohol ; Alg: Alginate; Gel: Gelatin; CMCs: Carboxymethyl Chitosan; CMC: Carboxymethyl cellulose; PAA: Polyacrilic acid; PVP: Polyvinyl pyrrolidone

**Table 3:** Contact angle of scaffolds, statistical comparison with PVA scaffold in ten seconds, and confidence interval comparison with human corneal tissue

***Sample***	**Average angle (°) **	**Confidence Interval (CI)**	**Is there a significant difference?**
*Polyvinyl alcohol *	44.44 ± 3.99	42.5 to 46.3	YES
*Alginate *	31.09 ± 2.51, *P* ˂ 0.0001	29.9 to 32.2	NO
*Carboxymethyl cellulose *	56.79 ± 5.03, *P* ˂ 0.0001	54.4 to 59.1	YES
*Carboxymethyl Chitosan *	64.15 ± 6.11, *P* ˂ 0.0001	61.2 to 67.0	YES
*Gelatin *	53.48 ± 2.63, *P* = 0.0007	52.2 to 54.7	YES
*Polyvinyl pyrrolidone *	44.65 ± 2.28, *P* = 0.9999	43.5 to 45.7	YES
*Polyacrilic acid *	70.53 ± 4.66, *P* ˂ 0.0001	68.3 to 72.7	YES
*PVA-Alg*	37.27 ± 4.57, *P* = 0.0085	35.1 to 39.4	NO
*PVA-CMC*	49.26 ± 3.78, *P* = 0.1320	47.4 to 51.02	YES
*PVA-CMCs*	49.61 ± 5.63, *P* = 0.0914	46.9 to 52.2	YES
*PVA-Gel*	46.79 ± 2.75, *P* = 0.8376	45.5 to 48.07	YES
*PVA-PVP*	43.12 ± 3.43, *P* = 0.9930	41.5 to 44.7	YES
*PVA-PAA*	56.53 ± 6.29, *P* ˂ 0.0001	53.5 to 59.4	YES

The values are presented as the mean ± SD, with a sample size of n = 3. ns = *P*˃0.05, *P<0.05, **P<0.01, ***P<0.001 and ****P<0.0001.

PVA: Polyvinyl alcohol ; Alg: Alginate; Gel: Gelatin; CMCs: Carboxymethyl Chitosan; CMC: Carboxymethyl cellulose; PAA: Polyacrilic acid; PVP: Polyvinyl pyrrolidone

**Table 4:** At 37 °C and various time points, the pH values of prepared scaffold. The values are presented as the mean ± SD, with a sample size of n = 3.

***Sample***	**0**	**2 hr**	**4 hr**	**6 hr**	**24 hr**	**48 hr**	**72 hr**
*Alginate *	7.13±0.09	7.10±0.15	7.12±0.10	7.17±0.14	7.22±0.10	7.28±0.04	7.29±0.04
*Carboxymethyl cellulose *	7.19±0.08	7.15±0.10	7.21±0.04	7.19±0.09	7.16±0.11	7.18±0.09	7.22±0.05
*Carboxymethyl Chitosan *	7.33±0.10	7.36±0.12	7.35±0.10	7.36±0.09	7.43±0.05	7.55±0.10	7.69±0.12
*Gelatin *	7.05±0.03	7.05±0.05	7.04±0.04	7.02±0.06	7.01±0.03	6.98±0.07	6.95±0.10
*Polyvinyl alcohol *	7.24±0.11	7.31±0.04	7.29±0.07	7.21±0.10	7.24±0.06	7.26±0.03	7.25±0.09
*Polyvinyl pyrrolidone *	6.90±0.10	6.94±0.07	6.94±0.05	6.94±0.06	6.93±0.07	6.87±0.05	6.86±0.07
*Polyacrilic acid *	6.12±0.08	6.01±0.10	6.00±0.03	5.98±0.05	5.83±0.06	5.34±0.09	5.31±0.03
*PVA-Alg*	7.22±0.03	7.21±0.05	7.20±0.05	7.20±0.04	7.19±0.02	7.18±0.04	7.17±0.06
*PVA-CMC*	7.18±0.07	7.19±0.04	7.19±0.07	7.19±0.06	7.20±0.05	7.25±0.08	7.29±0.10
*PVA-CMCs*	7.31±0.08	7.32±0.07	7.33±0.10	7.34±0.09	7.38±0.08	7.39±0.10	7.41±0.13
*PVA-Gel (50-50)*	7.15±0.06	7.15±0.05	7.14±0.06	7.14±0.04	7.10±0.03	7.08±0.06	7.03±0.01
*PVA-Gel (30-70)*	7.11±0.09	7.10±0.08	7.09±0.08	7.09±0.07	7.03±0.01	7.01±0.02	6.99±0.05
*PVA-Gel (70-30)*	7.21±0.02	7.19±0.09	7.20±0.05	7.19±0.06	7.18±0.02	7.17±0.07	7.14±0.09
*PVA-PVP*	7.03±0.09	7.02±0.01	7.00±0.05	7.00±0.04	6.99±0.02	6.95±0.06	6.92±0.04
*PVA-PAA*	6.57±0.12	6.52±0.09	6.52±0.12	6.50±0.04	6.42±0.06	6.38±0.09	6.31±0.10

PVA: Polyvinyl alcohol ; Alg: Alginate; Gel: Gelatin; CMCs: Carboxymethyl Chitosan; CMC: Carboxymethyl cellulose; PAA: Polyacrilic acid; PVP: Polyvinyl pyrrolidone

Significance levels are expressed as: ****P<0.0001, ***P<0.001, **P<0.01, and *P<0.05.

Values represent the mean ± SD, n = 3, *P<0.05, **P<0.01, and ns= non-significant. SD: standard deviation.

Values represent the mean ± SD, n = 3, *P<0.05, **P<0.01, and ***P<0.001. SD: standard deviation.

The values are presented as the mean ± SD, with a sample size of n = 3. The importance levels are represented by asterisks, where ****P<0.0001, **P<0.01, and *P<0.05. SD stands for standard deviation.

The values indicate the mean ± standard deviation, with a sample size of 3. Significance levels are denoted as **P<0.01 and *P<0.05.

Significance levels are expressed as: ****P<0.0001 compared to other groups. SD stands for standard deviation.

Values represent the mean±SD, n=3, *P<0.05, **P<0.01, ***P<0.001, and ****P<0.0001. SD, standard deviation.

**Table 5:** Evaluation of adhesion of cast scaffolds at different angles (0◦ to 120◦)

***Sample***	**0** ^◦^	**45** ^◦^	**90** ^◦^	**120** ^◦^	**Palm side**
*Alginate *	*	*	*	*	
*Carboxymethyl cellulose *	*	*	*	*	*
*Carboxymethyl Chitosan *	*	*	*		
*Gelatin *	*	*	*	*	*
*Polyvinyl alcohol *	*	*	*	*	
*Polyvinyl pyrrolidone *	*	*	*		
*Polyacrilic acid *	*	*			
*PVA-Alg*	*	*	*	*	
*PVA-CMC*	*	*	*	*	*
*PVA-CMCs*	*	*	*		
*PVA-Gel*	*	*	*	*	*
*PVA-PVP*	*	*	*	*	
*PVA-PAA*	*	*			

PVA: Polyvinyl alcohol ; Alg: Alginate; Gel: Gelatin; CMCs: Carboxymethyl Chitosan; CMC: Carboxymethyl cellulose; PAA: Polyacrilic acid; PVP: Polyvinyl pyrrolidone

Values represent the mean±SD, n=3, *P<0.05, **P<0.01, ***P<0.001, and ****P<0.0001. SD, standard deviation.

**Table 6:** Shows the methods of making wound dressings for the cornea using electrospinning, solvent casting, and 3D bio printers

***Method***	**Advantages**	**Disadvantages**
*Electrospinning*	Nanofibrous scaffolds, High surface area, control over porosity, Incorporation of bioactive molecules, and Biocompatible polymers	Difficult to scale for large-scale production, control over fiber alignment and diameter, not all polymers are suitable for electrospinning, and Solvent toxicity
*3D Bio-Printing*	Precise control over structure, multiple materials, or cell-laden bioinks to create layered structures, and incorporation of cells and growth factors	High cost, resolution limitations, and the complexity of performing the technique
*Solvent Casting*	Utilizes biodegradable materials, well-suited for large-scale production, with versatility for incorporating various bioactive molecules, and mimics ECM architecture	Solvent toxicity, control over scaffold porosity, and less precise for complex designs

## Conclusion

Corneal tissue engineering represents a pivotal advancement in regenerative medicine, addressing the urgent global burden of corneal diseases. With an estimated 4.2 million individuals affected by corneal blindness (WHO, 2023) and 1.5–2 million new annual cases of ulcers, trauma, and infections, the demand for innovative therapies is critical. This study focused on identifying transparent, biocompatible polymer scaffolds with corneal-appropriate mechanical strength. Through systematic evaluation of solvent-cast polymers—assessing biocompatibility, mechanical resilience, degradability, and optical clarity—we identified gelatin-polyvinyl alcohol (PVA) composites as optimal candidates. These blends synergize biological compatibility with robust physical-mechanical properties, closely mimicking native corneal tissue. Future work will integrate therapeutic agents (e.g., antimicrobial drugs, bioactive plant extracts) into these scaffolds to enhance regenerative efficacy. Such multifunctional dressings hold transformative potential for corneal repair, reducing reliance on donor tissues. This research advances biomaterial design and offers scalable solutions for ophthalmologists, tissue engineers, and biotech industries, ultimately alleviating the global burden of corneal blindness and improving patient outcomes.

## Declaration

We acknowledge the use of DeepSeek Open AI to improve language and readability in discussion section.
